# Gene Expression Profiles in Chemokine (C-C Motif) Ligand 21-Overexpressing Pancreatic Cancer Cells

**DOI:** 10.1007/s12253-018-0390-z

**Published:** 2018-04-23

**Authors:** Kai Cui, Hongsheng Zou, Mingliang Shi, Yang Ou, Lu Han, Bo Zhang, Dawei Hu, Sheng Li

**Affiliations:** 1 Shandong Cancer Hospital affiliated to Shandong University, Jinan, Shandong 250117 People’s Republic of China; 2 Shandong Academy of Medical Sciences, Jinan, Shandong 250200 People’s Republic of China; 3 People’s Hospital of Rongcheng, Rongcheng, Shandong 264300 People’s Republic of China; 4 People’s Hospital of Laiwu City, Laiwu, Shandong 271199 People’s Republic of China

**Keywords:** Chemokine (C-C motif) ligand 21, Pancreatic cancer cell, Lentiviral transduction, DNA microarray, Matrix metallopeptidase-9

## Abstract

Chemokine (C-C Motif) ligand 21 (CCL21) plays an important role in tumor immunity. However, the molecular mechanisms by which CCL21 regulates tumor immunity remain largely unknown. In this study, we successfully generated a lentiviral vector expressing human CCL21 (Lenti-hCCL21), which was confirmed by biological assays. The Lenti-hCCL21 was transduced into PANC-1 cells, a chemokine (C-C motif) receptor 7 (CCR7)-positive human pancreatic cancer cell line. We used the scratch wound and transwell assays to measure cell migration of the CCL21-overexpressing PANC-1 cells. A DNA microarray assay was performed to determine gene expression profiles. The results showed that CCL21 lentiviral transduction significantly up- or down-regulated a panel of tumor-associated genes, although CCL21 appeared to have no effect on PANC-1 cell migration. Importantly, CCL21 promoted matrix metallopeptidase-9 (MMP-9) expression in PANC-1 cells. CCL21 regulates pancreatic cancer immunity possibly through governing the expression of a panel of tumor-associated genes, including MMP-9.

## Introduction

Pancreatic cancer is the fifth leading cause of death from cancer worldwide [1]. It is a highly aggressive malignancy and the five-year survival rate is less than 5%. Currently available treatments provide only limited efficacy. Thus, there is an urgent need to develop new therapeutic strategies for pancreatic cancer.

Chemokines are a superfamily of small cytokines with chemoattractant properties that play a key role in chemotaxis, a process whereby cells migrate in response to a chemical gradient [2]. Chemokines mediate the host response to tumor by directing the trafficking of both anti-tumor and pro-tumor leukocytes into the tumor microenvironment [3]. Chemokine (C-C Motif) ligand 21 (CCL21) is a lymphoid chemokine that is abundantly and constitutively expressed by high endothelial venules in lymph nodes, lymphatic vessels, and stromal cells in the spleen and appendix [4]. CCL21, through binding to its receptor, chemokine (C-C motif) receptor 7 (CCR7), can attract dendritic cells (DCs), lymphocytes, natural killer (NK), and natural killer T (NKT) anti-tumor effectors to the tumor site. In addition, CCL21 stimulates these immune cells to produce cytokines such as interferon-γ, granulocyte-macrophage colony-stimulating factor, and interleukin-12, facilitating T cell activation and subsequently inhibiting tumor growth [5–7]. However, the molecular mechanisms or signaling pathways underlying CCL21-induced chemotaxis in cancer, especially in pancreatic cancer, remain largely unknown.

In this study, we transduced CCR7-positive PANC-1 cells with lentiviral vector expressing human CCL21 (hCCL21) to determine if CCL21 affects expression of certain genes associated with pancreatic cancer formation and development. Some genes, including matrix metallopeptidase-9 (MMP-9), were identified, providing new insight into CCL21 function in pancreatic cancer.

## Materials and Methods

### Cloning of hCCL21 cDNA

This study was approved by the Ethics Committee at Shandong Cancer Hospital Affiliated with Shandong University. Human lymph nodes were dissected during lymphadenectomy in gastric cancer patients enrolled at the same hospital. Written informed consent was obtained from all patients. Total RNA was extracted from the lymph nodes using Trizol reagent (Invitrogen, CA, USA). CCL21 cDNA was synthesized by reverse transcription-PCR (Fermentas, USA) using primer sets: 5’-GCT CTA GAA TGG CTC AGT CAC TGG CTC T-3′ (forward), 5′-ATA TGC GGC CGC CTA TGG CCC TTT AGG GGT CTG T-3′ (reverse). CCL21 cDNA was then inserted into pEASY-T1 simple cloning vector (Transgen Biotech, Beijing, China) and sequenced. The sequencing results were compared to the human CCL21 cDNA sequence reported in the GenBank database.

### Production of Lentiviral Vector Expressing hCCL21 cDNA (Lenti-hCCL21)

The cloned hCCL21 cDNA was digested with XbaI and NotI (MBI, USA) followed by ligation into pCDH-CMV-MCS-EF1-copGFP (SBI, CA, USA) using T4 DNA ligase (NEB, MA, USA). The product was transformed into Trans109 competent cells (Transgen Biotech, Beijing, China). The plasmids were prepared using the Mini RNeasy kit (Qiagen, Hilden, Germany) followed by recombination into the lentiviral vector FIV construction system (SBI, CA, USA). The recombinant plasmids were transfected into human embryonic kidney 293 (HEK-293) cells using Lipofectamine 2000 (Invitrogen, CA, USA) for lentiviral packaging. The supernatant was collected for preparation of lentiviral particles.

### Pancreatic Cancer Cell Lines

Five pancreatic cancer cell lines, AsPC-1, BxPC-3, CFPAC-1, PANC-1, and SW1990, were purchased from Cell Bank, Shanghai Institutes of Biological Sciences, Chinese Academy of Sciences, Shanghai, China, and cultured in Dulbecco’s modified Eagle medium (DMEM) supplemented with 10% fetal bovine serum (FBS) at 37 °C in a humidified atmosphere of 5% CO_2_. Total RNA was extracted from each cell line using Trizol reagent (Invitrogen, CA, USA), and subsequently reverse-transcribed for CCR7 and β-actin cDNA synthesis using the following primers: CCR7: 5′-TGA GGT CAC GGA CGA TTA CAT-3′ (forward), 5′-GTA GGC CCA CGA AAC AAA TGA T-3′ (reverse); β-actin: 5’-CCC AAG GCC AAC CGC GAG AAG AT-3′ (forward), 5’-GTC CCG GCC AGC CAG GTC CAG-3′ (reverse). The cDNA was amplified using the SYBR green real-time PCR kit (Roche, Basel, Switzerland) in the ABI7500 PCR system (Applied Biosystems, CA, USA).

### Lenti-hCCL21 Transduction in PANC-1 Cells

PANC-1 cells were plated at a density of 5 × 10^4^ cells in 10-cm dishes. Lenti-hCCL21 was added to each dish at multiplicity of infection (MOI) 10. Lenti-GFP was used as a negative control. Images were acquired using fluorescence microscopy.

### Scratch Wound Assay

PANC-1 cells were plated in a 6-well plate at a density of 1 × 10^5^ cells per well and grown at 37 °C and 5% CO_2_ for 48 h to form a confluent monolayer. Cells were then transduced with Lenti-hCCL21 or Lenti-GFP. To minimize the effect of cell proliferation on migration, the medium was replaced with fresh serum-free and mitomycin-containing (10 μg/mL) medium 2 h prior to scratch. The confluent monolayer of cells was scratched using a sterile micropipette tip to form a wound of 0.4–0.5 mm in width. The medium was immediately removed and replaced with fresh 10% FBS-containing complete medium. All scratch assays were performed in quadruplicate. At 24 h after scratch wound formation, images were captured with a Leica DMI4000B inverted microscope (Wetzlar, Germany) and the scratch width was measured.

### Transwell Assay

Cell migration assays were performed in a 24-well plate using 6.5 mm diameter transwell inserts with 8.0 μm pores. The upper side of the insert was thinly coated for 1 h with rat tail type I collagen. PANC-1 cells were resuspended in 100 μL of DMEM and loaded into the upper compartment filled with 600 μL of migration medium. Cells were allowed to migrate through the membrane of the inserts for 24 h at 37 °C and 5% CO_2_. The inserts were then washed with phosphate-buffered saline (PBS). The non-migrated cells remaining in the upper compartment were removed with a cotton swab. The migrated cells that adhered to the lower surface were fixed, stained, and counted visually under a light microscope at 40× magnification. Ten fields were randomly chosen and the mean number of cells was calculated for each group.

### DNA Microarray Assay

DNA microarray was performed using the RT^2^*Profiler* ™ PCR array (SuperArray, MD, USA). The expression levels of 84 tumor-associated genes were analyzed in blank, Lenti-GFP, and Lenti-CCL21 groups. Some microarray data were validated by Western blot analysis.

### Western Blot

PANC-1 cells were plated in 6-cm dishes at a density of 3 × 10^5^ cells per dish and grown at 37 °C and 5% CO_2_ overnight to form a monolayer. Cells were then transduced with Lenti-hCCL21 or Lenti-GFP for 72 h prior to protein extraction from culture medium as well as cells. 20 μg of proteins were separated by 10% SDS-PAGE and were transferred to PVDF membranes (Bio-Rad, CA, USA) followed by 2 h blocking with 5% bovine serum albumin (BSA) and overnight incubation with primary antibodies (Abs) against β-actin, CCL21, and MMP-9 (Cell Signaling, MA, USA) at 4 °C. The membranes were washed with PBS containing 0.05% Tween-20 (PBST) three times, and then incubated with HRP-conjugated secondary Abs for 1 h at room temperature followed by PBST washes. Chemiluminescent signal was detected on X-ray film. The film images were obtained using a gel imaging analysis system.

### Statistical Analysis

All experiments were repeated at least three times. Data are expressed as the mean ± standard error (SE). Statistical significance was assessed using Student’s *t* test or one-way ANOVA with SPSS16.0 statistical software (SPSS Inc., IL, USA). *p* < 0.05 was considered statistically significant.

## Results

### Cloning and Identification of hCCL21 cDNA

**Fig. 1:**
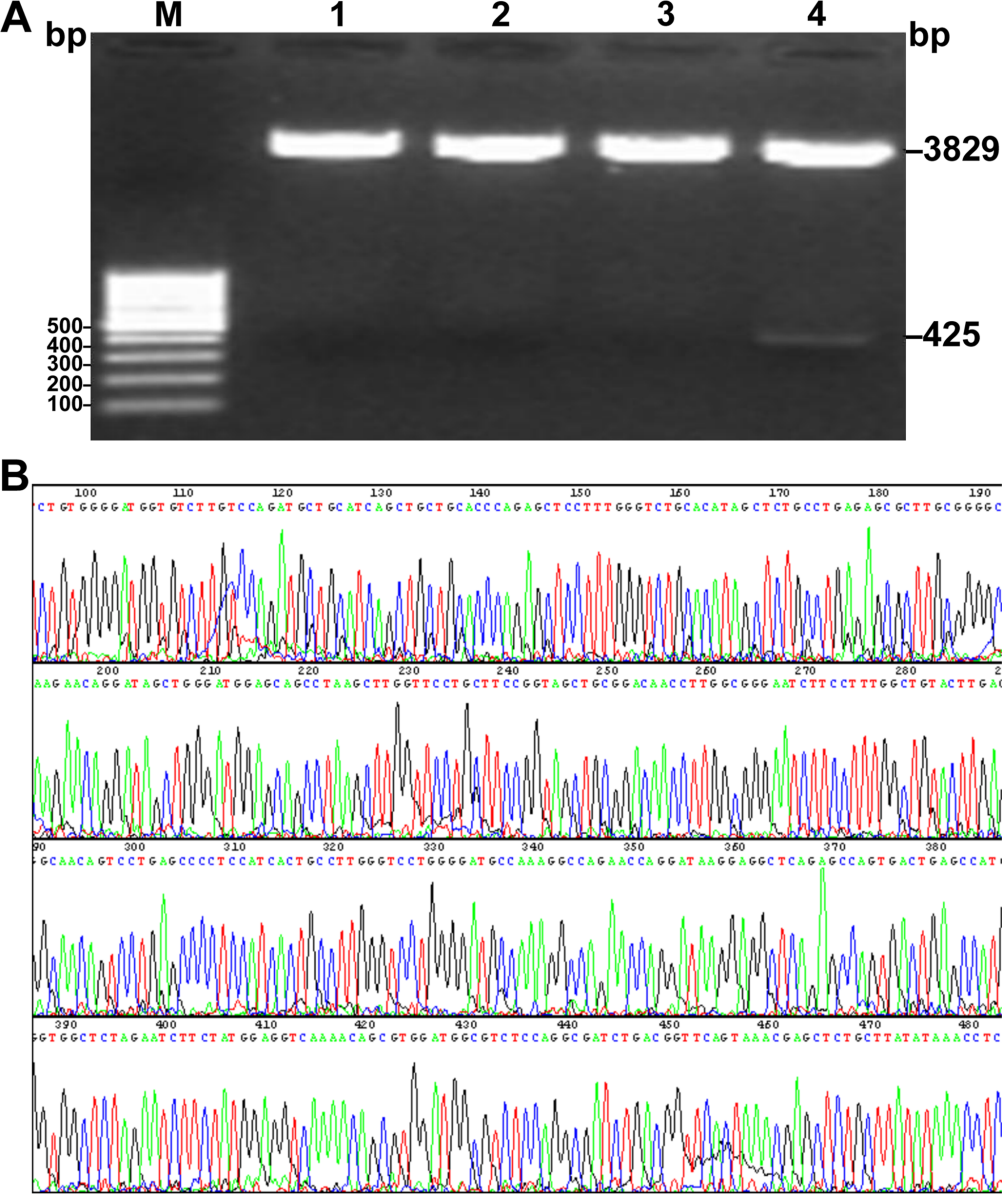
Cloning and identification of hCCL21 cDNA. **a** Agarose electrophoresis for undigested (lane 1–3) and digested (lane 4) hCCL21 cDNA cloning vector pEASY-T1 plasmids. M: DNA marker. **b** Full-length hCCL21 cDNA sequencing

To determine if hCCL21 cDNA was successfully cloned into pEASY-T1 simple-plasmids, we prepared plasmids from transformed bacteria and digested them with XbaI and NotI. Agarose electrophoresis results showed a band between 400 and 500 bp following enzymatic digestion (Fig. 1a), consistent with the size of hCCL21 cDNA (425 bp) reported in the GenBank database. The sequencing results also confirmed that the cloned fragment was identical to the hCCL21 cDNA sequence (Fig. 1b), suggesting that hCCL21 cDNA was successfully cloned into the vector without any mutations.

### Successful Transduction of Lentiviral Vector Expressing CCL21 in PANC-1 Cells

**Fig. 2:**
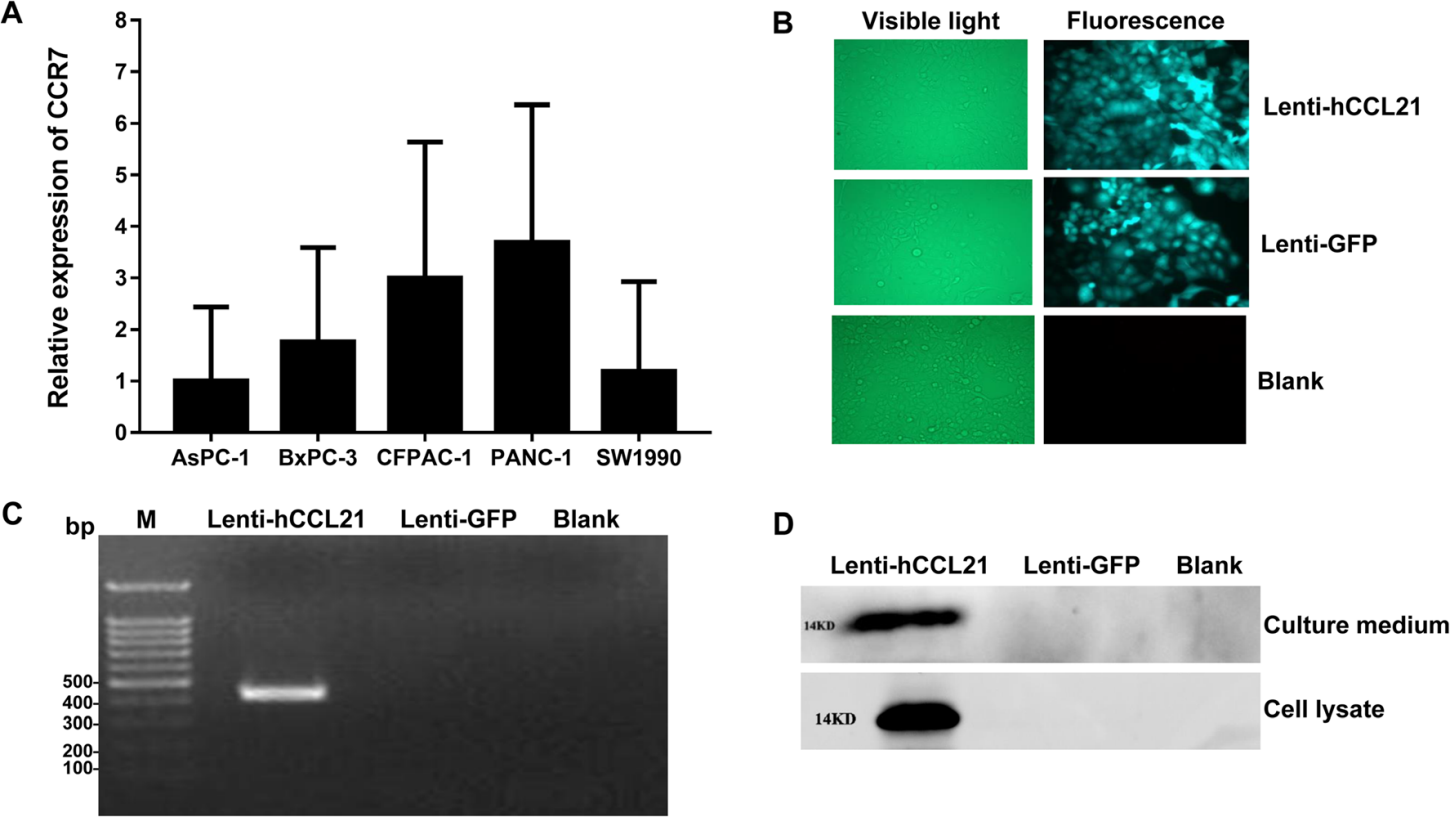
Successful transduction of PANC-1 cells with lentiviral vector expressing CCL21 (Lenti-hCCL21). **a** Quantitative real-time PCR analysis of CCR7 expression in five pancreatic cell lines. M: DNA marker, *n* = 3. **b** Fluorescent detection of lentiviral transduction. Magnification: × 200. **c**. Reverse transcription and PCR analysis of CCL21 expression in PANC-1 cells. M: DNA marker. **d** Western blot assay for CCL21 expression and secretion in lentiviral-transduced PANC-1 cells

PANC-1 cells were selected as the target cell line since these cells had the highest expression of the CCL21 receptor, CCR7, relative to the five other pancreatic cell lines (Fig. 2a). Furthermore, fluorescent imaging showed that green fluorescence appeared in almost all of the PANC-1 cells transduced with GFP- and CCL21-expressing lentiviral particles (Fig. 2b), suggesting high transduction efficiency of the prepared lentiviral vector in PANC-1 cells. Importantly, CCL21 mRNA and protein expression were dramatically up-regulated in CCL21-expressing lentiviral vector-transduced PNAC-1 cells compared to the control GFP-expressing lentiviral vector and the blank groups (Fig. 2c and d). In addition, we also detected CCL21 protein expression in the culture medium of PANC-1 cells transduced with the CCL21-expressing lentiviral vector (Fig. 2d). These results suggest that the CCL21-expressing lentiviral vector was successfully transduced into PANC-1 cells, leading to high-levels of CCL21 protein expression and secretion.

### Effects of CCL21 on PANC-1 Cell Morphology and Migration

**Fig. 3:**
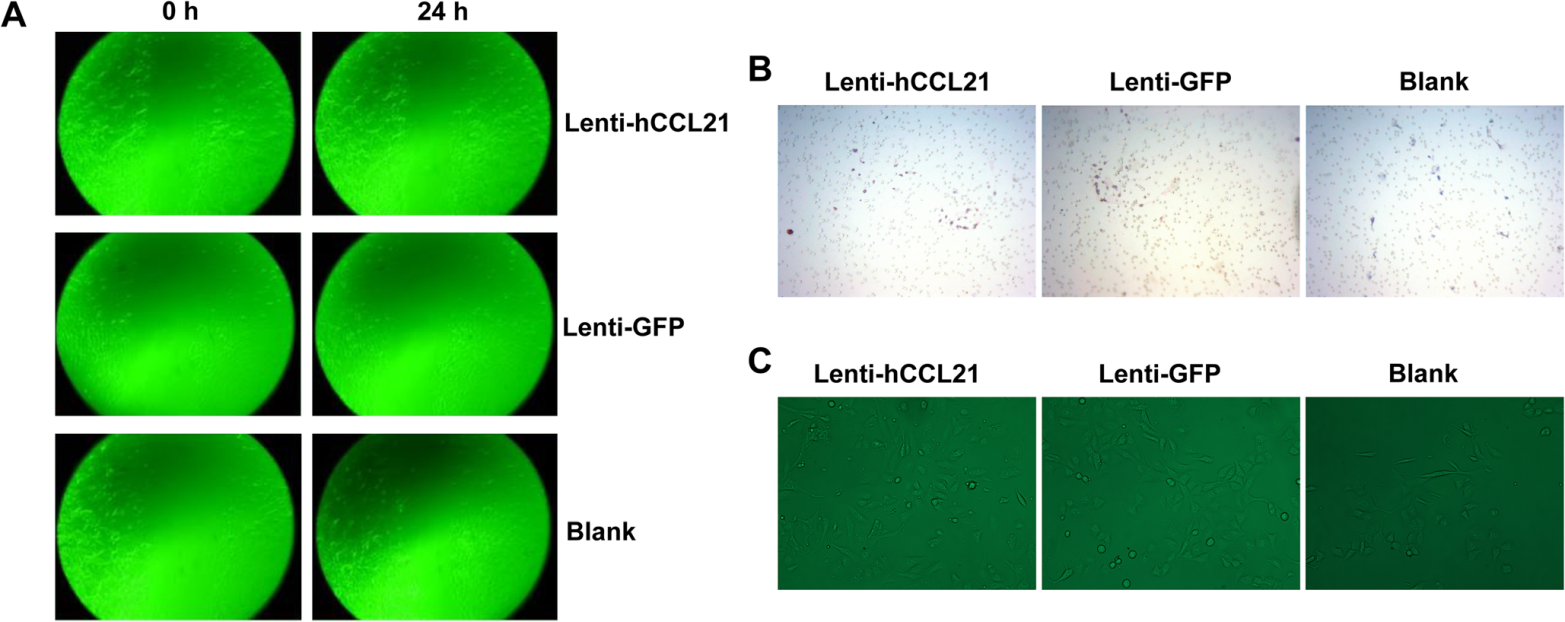
CCL21 had no effect on PANC-1 cell migration and morphology. **a** Scratch wound assay for PANC-1 cell migration. **b** Transwell assay for PANC-1 cell migration. **c** Microphotographs showing PANC-1 cell morphology

To determine the function of CCL21 in PANC-1 cells, we performed a scratch wound assay. CCL21 had no effect on PANC-1 cell migration (Fig. 3a and Table 1), suggesting that CCL21 does not inhibit the in vitro metastatic ability of PANC-1 cells. Similar results were also observed using the transwell assay (Fig. 3b and Table 2). Furthermore, there was no significant difference in cellular morphology between the control and CCL21-expressing lentiviral vector groups (Fig. 3c), suggesting that CCL21 does not regulate PANC-1 cell differentiation.

**Table 1:** Quantification of cell migration shown in A

Groups	Field numbers	Scratch width (*x* ± *s,* mm)
Lenti-hCCL21	10	4.53 ± 0.42
Lenti-GFP	10	4.47 ± 0.15
Blank	10	4.3 ± 0.26

*P* > 0.05 between any two groups

**Table 2:** Quantification of cell migration shown in B

Groups	Field numbers	Numbers of transwell cells (*x* ± *s*)
Lenti-hCCL21	10	30.15 ± 4.35
Lenti-GFP	10	28.68 ± 4.16
Blank	10	27.95 ± 3.99

*P* > 0.05 between any two groups

### Effects of CCL21 on the Expression Profile of Tumor-Associated Genes

**Fig. 4:**
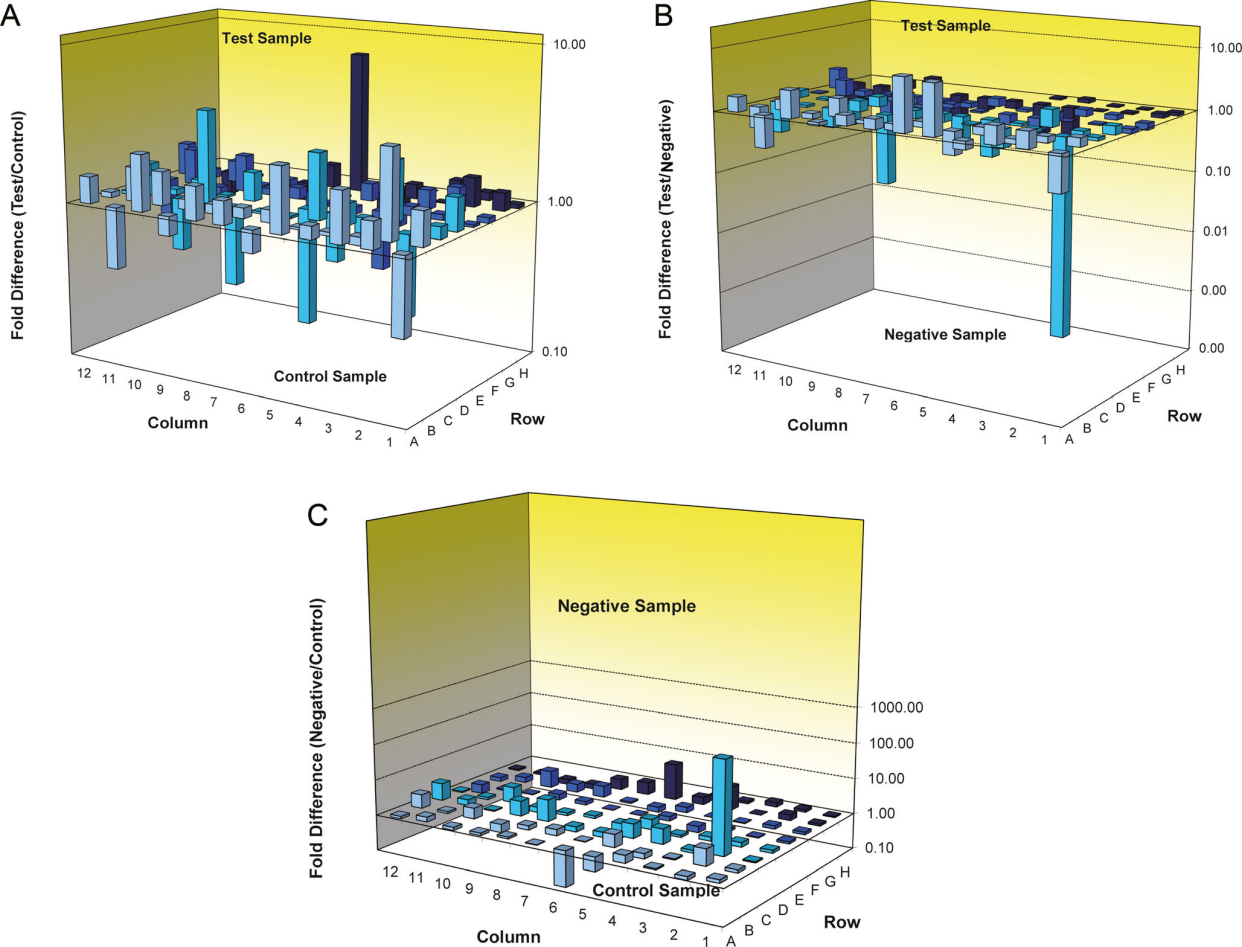
Relative mRNA expression levels in Lenti-hCCL21-transduced PANC-1 cells, compared to (**a**) Lenti-GFP-transduced and (**b**) non-transduced groups; **c** Relative mRNA expression levels in non-transduced groups compared to Lenti-GFP-transduced groups

**Fig. 5:**
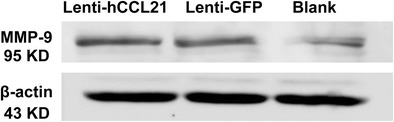
Western blot assay for the effect of CCL21 on MMP-9 expression in PANC-1 cells

Since CCL21 did not play a role in in vitro metastasis and differentiation of PANC-1 cells in this study, we next sought to determine if CCL21 has potential effects on other aspects of PANC-1 cells. We identified a panel of tumor-associated genes that were either significantly upregulated or downregulated by more than 2-fold in CCL21-overexpressing PANC-1 cells in our DNA microarray analysis (Table 3). The identified genes are involved in cell cycle (ataxia- telangiectasia mutated (ATM) and breast cancer 1 (BRCA1)), apoptosis (caspase 8 (CASP8)), cell adhesion (alpha-2 integrin (ITGA2)), angiogenesis (interleukin 8 (IL-8)), and cell signaling pathways (involving serine-threonine protein kinase (AKT1), Fos proto-oncogene (FOS) and Jun proto-oncogene (JUN)). Previous studies revealed that the tumor vasculogenesis-associated gene MMP-9 is clinically important in pancreatic cancer growth and metastasis [8, 9], although MMP-9 is not among the identified genes in our microarray analysis. To examine if there is a correlation between CCL21 and MMP-9, we performed Western blot analysis and demonstrated that CCL21 promoted MMP-9 protein expression in PANC-1 cells (Fig. 5), suggesting that CCL21 plays a potential role in vasculogenesis and vascularization in pancreatic cancer. Figure 4 shows fold change in mRNA expression levels among Lenti-hCCL21-transduced, Lenti-GFP-transduced, and non-transduced PANC-1 cells, based on the results of the RT^2^*Profiler*™PCR microarray.

**Table 3:** RT^2^*Profiler*™PCR microarray analysis of CCL21-regulated genes

Gene	Functions	Lenti-hCCL21	Lenti-GFP	Blank
ATM	Cell cycle regulation	6.46	2.6	−2.49
BRCA1	Cell cycle regulation	2.8	2.31	−1.22
CASP8	Apoptosis	−3.36	−2.48	1.35
AKT1	Signaling transduction and transcription	−3.82	−3.1	1.23
FOS	Signaling transduction and transcription	−1225.31	−3.26	375.71
JUN	Signaling transduction and transcription	−3.82	−2.02	1.89
ITGA2	Cell adhesion	−2.05	−2.14	−1.05
IL8	Angiogenesis	−12.77	−3.16	4.05

## Discussion

CCL21 plays important roles in tumor apoptosis, infiltration, metastasis, and angiogenesis [10–12]. Therefore, targeting CCL21 is a potential therapeutic intervention in cancers, including pancreatic cancer. In the present study, we successfully generated lentiviral vectors expressing hCCL21, which resulted in dramatically increased expression and secretion of CCL21 in the pancreatic PANC-1 cell line (Fig. 2). Consistent with previous findings [13], our data indicate that endogenous expression of CCL21 is undetectable in PANC-1 cells (Fig. 2), suggesting a possible protective role of CCL21 in pancreatic cancer cells. Unexpectedly, CCL21-overexpressing PANC-1 cells did not exhibit decreased migration ability compared to the control group (Fig. 3). A possible explanation for this paradoxical result is that CCL21 itself is not sufficient to inhibit tumor cell migration, and additional factors may be required for CCL21 to regulate PANC-1 cell migration. Another possible reason is that the in vitro experimental environment in this study differs from the in vivo tumor microenvironment where pancreatic cancer cells reside, leading to inadequate responses of CCL21 to microenvironmental cues. Consequently, the role of CCL21 in pancreatic cancer cell migration and metastasis needs to be reassessed in an in vivo study, which requires further investigation.

Our data indicate that CCL21 does not suppress pancreatic cancer cell migration; however, the DNA microarray data demonstrate that CCL21 can regulate expression of certain tumor-associated genes (Table 3). Among these genes, cell cycle-related genes like ATM and BRCA1 were significantly upregulated by CCL21, suggesting that CCL21 may contribute to PANC-1 cell proliferation. Consistent with this result, CCL21 decreased expression of the pro-apoptotic gene, CASP8. In contrast, CCL21 inhibited expression of signaling transduction molecules and transcription factors AKT1, FOS and JUN, as well as the angiogenic cytokine IL-8 and adhesion molecule ITGA2. Based on these data, CCL21 seems to have both anti- and pro-tumor effects on pancreatic cancer cells. Various lines of evidence also support the dual roles of CCL21 in pancreatic cancer development [14–18]. This likely depends on the cell line-based model system of pancreatic cancer and the experimental conditions used in these studies.

MMP-9 is an important gene involved in pancreatic cancer growth and metastasis [19]. Our DNA microarray data show that CCL21 induced a modest increase in MMP-9 mRNA expression (less than 2-fold), consistent with CCL21-induced MMP-9 protein expression (Fig. 5). The correlation between CCL21 and MMP-9 in PANC-1 cells may provide valuable insights into molecular mechanisms underlying the functions of CCL21 in pancreatic cancer. Furthermore, our study identifies some CCL21-regulated genes that have potential clinical applications as diagnostic and prognostic biomarkers in pancreatic cancer.

*AKT1*, serine-threonine protein kinase; *ATM*, ataxia telangiectasiamutated; *BRCA1*, breast cancer 1; *BSA*, bovine serum albumin; *CASP8*, caspase 8; *CCL21*, Chemokine (C-C Motif) ligand 21; *CCR7*, chemokine (C-C motif) receptor 7; *DCs*, dendritic cells; *FOS*, Fos proto-oncogene; *IL-8*, interleukin 8; *ITGA2*, alpha-2 integrin; *MMP-9*, promoted matrix metallopeptidase-9; *NK*, natural killer; *NKT*, natural killer T; *PBS*, phosphate-buffered saline.
